# Dissociated Recombinant Human Bone Morphogenetic Protein-2 (rhBMP-2) Retains Osteoinductive Potential: An In Vivo Proxy for Burst Release

**DOI:** 10.7759/cureus.113014

**Published:** 2026-07-20

**Authors:** Elisa Tarsitano, Charles S Matthews, Mathieu Riffault, Malo Daniel, Florent Anavoizat, Souad Adrouche, Christelle Moal, Sejal Odedra, John P Von Benecke

**Affiliations:** 1 Research, Locate Bio Ltd, Nottingham, GBR; 2 Research, Atlantic Bone Screen, Saint-Herblain, FRA

**Keywords:** burst release, ectopic bone, intermuscular implantation, in vivo rat model, osteoinduction, rhbmp-2

## Abstract

Introduction

This study investigates whether recombinant human bone morphogenetic protein-2 (rhBMP-2), once dissociated from its carrier (absorbable collagen sponge (ACS)) and transported as a soluble factor, retains sufficient osteoinductive capacity to induce ectopic bone formation.

Methods

ACSs were loaded with rhBMP-2 (1.5 mg/mL) and centrifuged at 500 rcf to generate supernatant run-off (SRO) containing dissociated rhBMP-2. The concentration of rhBMP-2 in the SRO was quantified by UV spectrophotometry (n = 9). In a paired design, 10 athymic nude rats received intermuscular implantation of the centrifuged ACS in the right hindlimb and injection of the corresponding SRO into the contralateral left hindlimb. At 28 days, implant sites were evaluated by micro-CT for bone volume (BV), tissue volume (TV), bone volume fraction (BV/TV), and bone mineral density (BMD), followed by histological assessment.

Results

In vitro quantification determined that centrifugation released an average of 11.3% ± 6% of the rhBMP-2 load into the SRO. In vivo micro-CT analysis at 28 days demonstrated mineralised tissue formation at both sites. The SRO group exhibited significantly lower total tissue volume than the ACS group (8.05 mm³ vs 29.4 mm³, P < 0.05). No statistically significant differences in bone volume fraction (BV/TV) or bone mineral density (BMD) were detected between groups. Histological evaluation confirmed that mineralised tissue variably represented bone, with the SRO group showing a spectrum from mineralised cartilage to woven bone to corticocancellous-like architecture. The ACS group demonstrated more consistent bone formation but with greater heterogeneity in maturation, including cystic and adipose-dominant phenotypes in some animals. No correlation was observed between the volume of SRO administered and the resulting mineralised tissue volume.

Conclusions

rhBMP-2 that dissociates from its delivery scaffold retains osteoinductive potential and can induce mineralised tissue when transported as a soluble factor. Histologically, this mineralisation ranged from mineralised cartilage to mature bone. Since clinical burst release substantially exceeds the 11% tested here, these findings represent a conservative estimate of the clinical risk. Liberated rhBMP-2, even in small quantities, may contribute to off-target mineralisation, which could be clinically relevant if formed adjacent to neural elements. These findings emphasise the need for improved controlled-release delivery strategies.

## Introduction

Bone morphogenetic protein-2 (BMP-2) is a homodimeric growth factor in the transforming growth factor-β (TGF-β) superfamily that plays a pivotal role in bone formation and repair [1,2]. Clinically, recombinant human BMP-2 (rhBMP-2) is widely used in spinal fusion procedures and fracture healing [3]. This study investigates whether rhBMP-2 rapidly released from an absorbable collagen sponge (ACS) retains osteoinductive potential and can induce ectopic mineralisation in a rat model after dissociation from its carrier.

Despite its potent osteoinductive capacity, a major challenge is the poor retention of rhBMP-2 when delivered on an ACS at implantation sites [4]. This short retention necessitates high initial doses to ensure sufficient rhBMP-2 is present when the critical cell density for bone formation is reached [5]. This supraphysiological dosing approach has limitations, and there is clear evidence of a dose-response relationship with side effects [6]. Reported localised side effects include osteolysis, inflammation, and adipogenesis [7]. Ectopic bone formation in proximity to the implant site has also been reported clinically [7], occurring in ~13.5% of patients, but mostly without clinical sequelae [8].

James et al. [7] argue that ectopic bone formation may occur clinically due to premature leakage of rhBMP-2 during manual manipulation of the ACS. In contrast, Hsu et al. [9] evaluated the loss of rhBMP-2 during typical surgical handling and found that the supernatant of compressed ACS did not produce ectopic bone when subsequently implanted in rodents. Rather than handling, the mechanistic explanation for this short in vivo retention can be explained by the orders-of-magnitude lower binding affinity of surface-attached rhBMP-2 to delivery materials [10,11] relative to its much higher binding affinity for alternative in vivo binding sites, such as cells [12], BMP-2 antagonists [13-15], and extracellular matrix components [16]. For example, the association constant (Ka) of BMP-2 to collagen is reported to be 10³ to 10⁴ M⁻¹, whereas its dissociation constant (Kd) for its cellular receptor is measured at one to two nM. This is equivalent to a 50,000- to 1,000,000-fold difference, causing the BMP-2 to be quickly stripped away in the presence of in vivo cells and proteins. This explains the dramatically contrasting results reported when measuring the release profile of BMP-2 using ELISA in vitro and subsequently fluorescently labelled BMP-2 in vivo [17].

rhBMP-2 retention and transportation

The retention of rhBMP-2 has been studied by various authors and shows a relatively short retention profile in vivo [4,18]. For example, when rhBMP-2 was applied to an ACS and implanted into a metaphyseal defect in a non-human primate, just 47.1% (± 1.5%) was retained after 30 minutes, and 24.2% (± 3.5%) at one week [4].

Once the BMP-2 detaches from the carrier material, it can migrate away. Clinically, this is well demonstrated in provocative discography: when a contrast dye is injected into a damaged disc, it will leak through annular fissures into adjacent spaces. It is the extent of contrast extravasation seen on coronal CT imaging that is used to grade the severity of an annular tear [19].

This phenomenon confirms that if a patent pathway exists between the disc space and surrounding tissue, liquids and soluble factors will follow the path of least resistance and migrate out. By analogy, during interbody fusion surgery, part of the annulus is removed to insert a cage, and any solubilised BMP that becomes detached from its carrier can similarly seep out through the surgical defect. Once dissociated from the delivery material, the supraphysiological localised dose creates a BMP-2 concentration gradient along which it can disperse into adjacent tissues by passive diffusion [20] and interstitial fluid bulk flow [21]. This means biologics may not remain strictly in the disc space post-implantation, and some portion may diffuse into the epidural space, along muscle planes, or around nerve roots.

In sheep interbody studies, significant localised adverse effects, including osteolysis [6] and bone cysts [22], are reported when rhBMP-2 is delivered on ACS, with side effects peaking around three to four weeks post-implantation [6]. The models have consistently found a dose-response relationship with adverse events [6,23]. In the same model, Christou et al. evaluated the chimeric protein containing rhBMP-2, called AMP2 (Osteoadapt SP, Theradaptive), and reported partial paralysis in 2 of 18 (11%) high-dose AMP2 animals, also at the three- to four-week time point [24]. In those animals, the CT scan before euthanasia confirmed good placement of the interbody cage, with osteolysis and remodelling of the endplates, and several small dense foci encroaching slightly into the vertebral canal. Whilst the authors did not propose a cause for this ectopic bone, the time course of this presentation, well after the intraoperative or early postoperative period (zero to three days), suggests it is rhBMP-2-related rather than iatrogenic. Thus, the potential for ectopic bone growth caused by the rapid loss of retention from the scaffold, sometimes referred to as "drug burst", is investigated in this study.

To replicate the rapid release of rhBMP-2 seen in vivo [4,18], we utilised a centrifuge to dissociate a portion of the rhBMP-2 from the ACS. The ACS was implanted into the right leg muscle of rodents. To mimic the intercellular transport of the rhBMP-2, the supernatant run-off (SRO) dissociated from the ACS was implanted into the contralateral leg muscle, and both sites were assessed for bone formation at 28 days.

## Materials and methods

In vitro experiment

Absorbable collagen sponges (ACS; Medtronic - Cat. No. 7510200, Lot MLE4545AAJ) were sectioned into standardised dimensions of 1.25 cm × 0.7 cm × 0.2 cm (length × width × depth) to mirror the volume used in the corresponding in vivo implantation model. Each sponge segment was placed into an 8-μm cell culture insert, which was subsequently positioned at the bottom of a 50 mL conical centrifuge tube. Ten sponge samples were loaded with 98 μL of reconstituted Infuse rhBMP-2 (1.5 mg/mL, buffer as supplied with the product), applied dropwise to ensure uniform distribution across the ACS, providing 147 μg per sponge. Two additional sponges served as controls and received 98 μL of Infuse formulation buffer only. The sponges were incubated at room temperature for 15 minutes, following the manufacturer's instructions for use (IFU), thus simulating clinical preparation protocols. After incubation, all samples were centrifuged at 500 rcf for five minutes to simulate the release of rhBMP-2. The resulting SRO was carefully collected from the bottom of each tube and transferred to labelled 1.5 mL microcentrifuge tubes.

The concentration of rhBMP-2 in the SRO was quantified by measuring absorbance at 280 nm using ultraviolet (UV) spectrophotometry and interpolated based on a standard curve of known dose concentrations (n = 8, using two-fold serial dilutions down from 1.5 mg/mL). Absorbance readings were corrected by subtracting values obtained from collagen-only controls (ACS with buffer alone) to account for any background signal from the carrier matrix. This enabled the determination of the quantity of rhBMP-2 within the SRO.

In vivo model

The model used for this study is in line with the European Directive 2010/63/UE, September 22, 2010. Use of animals was approved by the ethical review committee (Comité d'Ethique en Expérimentation Animale des Pays de la Loire n°6), and ethical approval was granted by the French Ministry for Higher Education, Research and Innovation (ethical authorisation number: APAFIS#26526).

We utilised the guidance from ASTM standard F2529-13 for the in vivo evaluation of osteoinductive potential and used an athymic nude rat model to evaluate bone induction without interference from an immune response to human proteins. A total of 10 male Hsd:RH-Foxn1rnu rats (Envigo, eight to nine weeks old) were included. The sample size was set to the minimum required to adequately conduct the experiment, consistent with other studies [25]. Animals were acclimatised for 12 days prior to the beginning of the study, and during that time, their general clinical state was monitored daily. After surgery, an in-depth follow-up was performed twice a week with detailed clinical examination and body weight evaluation.

Animals were housed in dedicated polypropylene individually ventilated cages of standard dimensions with a maximum of three animals per cage. The housing maintained appropriate and stable temperature as well as humidity for the welfare of the animals. Moreover, an artificial day/night light cycle was set to 12 hours of light and 12 hours of darkness. All animals had free access to water and were fed ad libitum with commercial chow.

The ACS from an Infuse Bone Graft (Medtronic) kit was aseptically cut into pieces measuring 1.25 cm × 0.7 cm × 0.2 cm (l × w × d) to produce the required implant volume of 0.175 cc. BMP-2 solution was prepared as per the IFU, and, using a micropipette and a sterile tip, 98 µL of rhBMP-2 solution was added to each ACS piece. These were left for 15 minutes as per the manufacturer's IFU, and then each sponge was placed in a 0.8 μm cell culture insert, which was subsequently positioned at the bottom of a 50 mL conical centrifuge tube. The ACS pieces were then placed in a centrifuge and spun at 500 rcf (g) for five minutes. The SRO was collected in a labelled 1.5 mL tube, ensuring that the correspondence between the liquid and sponge was maintained. While maintained at room temperature, both the ACS and the corresponding SRO were transferred to the operating room and implanted within two hours.

Surgical procedure

Anaesthesia was induced with 5% isoflurane in air (0.5 L/min) and maintained at 2%-3%. Buprenorphine (0.05 mg/kg, Axience) was administered subcutaneously 30 min preoperatively, 4.5-6.25 h postoperatively, and on the following day. Lidocaine (MSD) was applied before the skin incision. Animals received preoperative subcutaneous 0.9% saline (Cooper) for rehydration, and eye protection was provided with Ocrygel. Additionally, rehydration was provided to each animal during the preoperative procedure by subcutaneous injection of saline.

A longitudinal skin incision was made parallel to the femur, midway between the hip and knee. The membrane between the biceps femoris and gluteus superficialis was punctured, and a pocket was created between the muscle groups without damaging the fibres.

Since each animal would represent its own control, with both SRO and ACS implants in the same animal, and given the uniform species, gender, and weight of the animals, no randomisation was performed. While laterality was not randomised (ACS right, SRO left), bilateral symmetry of rodent hindlimbs has been well established. Mustafy et al. demonstrated that left and right rat tibiae exhibit no significant differences in mechanical, geometrical, or morphological parameters, validating the use of the contralateral limb as an internal control [26]. This bilateral symmetry, combined with the paired design and systemic distribution of any circulating factors, indicates that laterality bias is negligible in this rat model. In the right leg, the ACS was inserted, and the pocket was sutured to prevent migration. In the left leg, the SRO (from the same ACS) was pipetted into the pocket. The subcutaneous and skin layers were closed with sutures. At day 28, rats (n = 10) were euthanised via cervical dislocation under isoflurane anaesthesia.

Radiographic assessment

All implant sites were analysed by micro-CT. Micro-CT acquisition was performed on a high-resolution X-ray micro-CT system for small-animal imaging (SkyScan 1076, Bruker microCT, Kontich, Belgium), with the following parameters: source voltage, 70 kV; rotation step, 0.6°; voxel size, nine μm³; one frame per position. Reconstruction of CT scan images was performed by generating a series of two-dimensional binarised cross-sections from the acquisition data using NRecon software (Version 1.7.4.6). For analysis, images were reoriented according to the central axis of radiopaque structures in three dimensions using DataViewer software (Version 1.6.0.0).

The volumes of radiopacity, identified as bone volume (BV), total volume (TV), and bone mineral density (BMD), were determined using CTAn software (Version 1.20.8.0, Bruker microCT, Kontich, Belgium). Threshold values for mineral tissue were 20-255. Because threshold-based micro-CT segmentation quantifies radiopaque mineralised tissue, the term "bone volume" in this analysis represents a mineralised tissue proxy; histology was used as the reference method for confirming true bone formation. Results were assessed by an independent reviewer who, due to the use of non-randomised limbs, was not blinded to the treatment groups.

Data are presented as mean ± standard deviation (SD). Given the paired study design, in which each animal received ACS in the right hindlimb and SRO in the contralateral left hindlimb, the non-parametric Wilcoxon signed-rank test was used to compare micro-CT parameters between groups. This test was selected because of the small sample size (n = 10 pairs) and because it does not assume normality of the paired differences. Effect size was calculated using the formula r = Z/√n, where values of 0.1, 0.3, and 0.5 represent small, medium, and large effects, respectively. Statistical significance was set at p < 0.05. All analyses were performed using Python (SciPy v1.11, SciPy Community, NumFOCUS, Austin, Texas).

Histological assessment

Ectopic tissues were fixed in 10% neutral-buffered formalin for 14-28 h (RT) and decalcified in 15% EDTA/PBS for 40 days with gentle agitation. Decalcification was monitored by radiography (Faxitron MX20, Edimex, Angers, France). Samples were rinsed, dehydrated, and cleared using an automated processor (ASP6025, Leica Biosystems, Nussloch, Germany) through graded ethanol (70% for 40 min; 80% for 40 min; 96% for 40 min; 100% for 60 + 120 + 120 min at 37°C), xylene (45 + 90 + 90 min at 37°C), and molten paraffin (60 + 90 + 120 min at 57°C). Tissues were embedded in Histostar (Thermo Fisher, Waltham, Massachusetts) and sectioned at 4 μm (Histocore Autocut, Leica Biosystems). Sections were floated on a 48°C water bath, mounted on Superfrost Plus slides, and dried overnight at 60°C. Slides were stained with haematoxylin and eosin (Spectra ST autostainer, Leica) and digitised at 20× magnification (Nanozoomer S60, Hamamatsu Photonics K.K., Hamamatsu, Japan). Results were reviewed by a single blinded reviewer.

## Results

In vitro quantification of dissociated SRO rhBMP-2

The concentration of rhBMP-2 in the SRO from the 10 rhBMP-2-treated sponges was quantified by measuring absorbance at 280 nm using ultraviolet (UV) spectrophotometry and interpolated to known dose concentrations to determine the amount of rhBMP-2. One sample yielded insufficient supernatant volume (2 µL) and was excluded from analysis.

**Figure 1:**
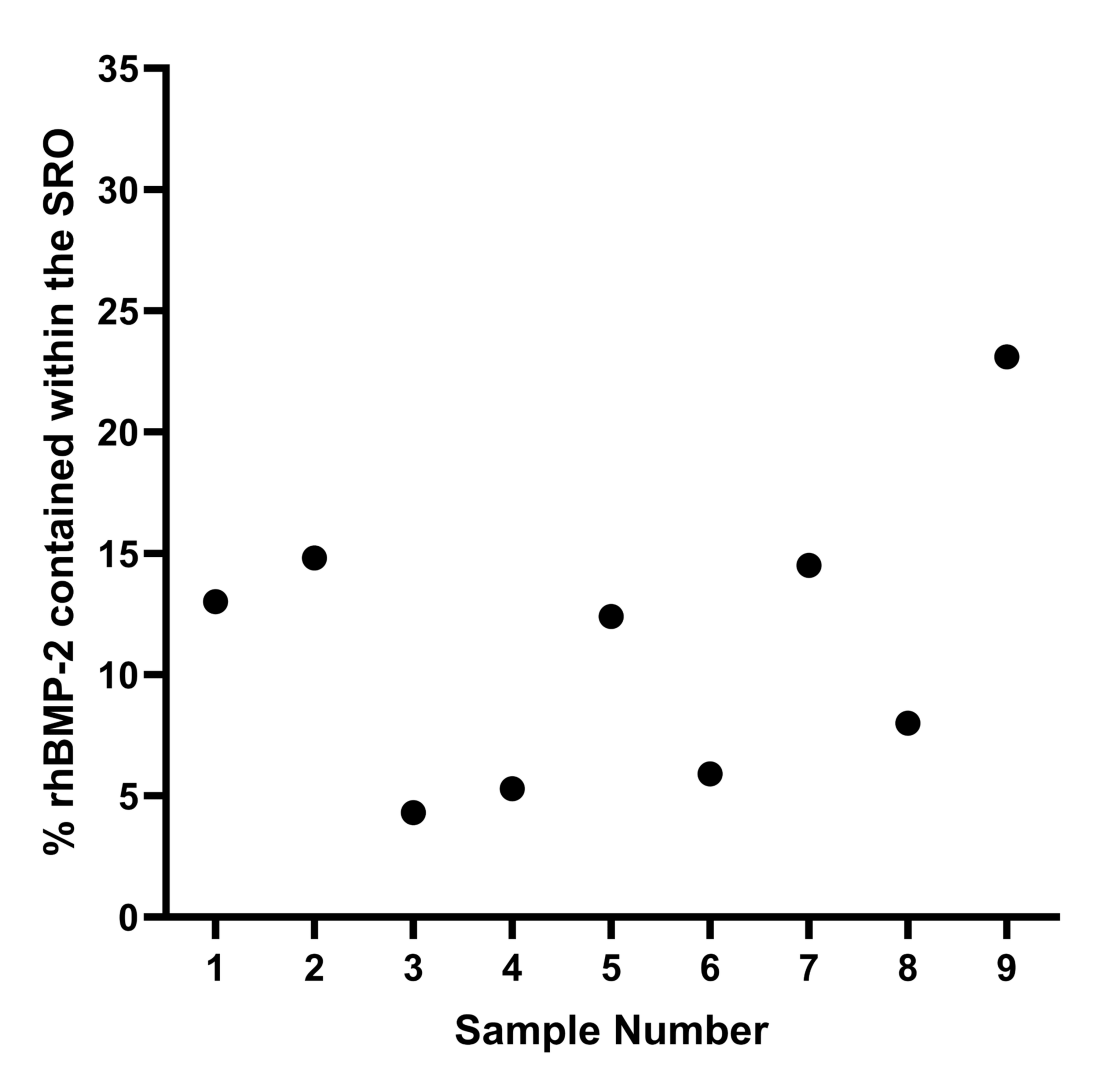
The amount of rhBMP-2 recovered from the SRO per sample, expressed as a percentage of the total rhBMP-2 per sample pre-centrifuge One sample yielded insufficient supernatant volume (2 µL) and was excluded from analysis. The average rhBMP-2 quantified in the SRO was 11.3% ± 6%. SRO: supernatant run-off, rhBMP-2: recombinant human bone morphogenetic protein-2.

Of the remaining nine samples, an average of 64.6 µL (44-74 µL) of the 98 µL of solution was recovered after the centrifugation step, as reported in Figure 1. The average rhBMP-2 quantified in the SRO was 11.3% ± 6%. There was a moderate but non-significant Pearson correlation (r = 0.47, p = 0.20) between the volume of liquid recovered and the amount of rhBMP-2 in the SRO. The amount of rhBMP-2 recovered is consistent with the data reported by Hsu et al. [9] for their Group 2 compression treatments. The authors reported a release of BMP-2 under compression of 10% (± 9%) for this group, within a range of volumes between 21% and 46% of the starting liquid volume.

It is noteworthy that the results from our simulated release using a centrifuge are substantially less than the 53% loss reported by Seeherman et al. [4] after 30 minutes following implantation into a proximal femoral core defect model in non-human primates. Thus, the centrifugation step is conservative relative to the initial rapid release of BMP-2 from ACS demonstrated in that model. 

In vivo results

The volume of SRO collected from each of the 10 samples prior to implantation is shown as a percentage of the total liquid applied to the ACS in Table 1. The average was 72.5 µL (74.0%).

**Table 1:** SRO volume after centrifuge for in vivo experiment SRO: supernatant run-off.

Animal Number	Volume of SRO (μL)	% of 98 µL
374	75	76.5
375	65	66.3
376	65	66.3
377	71	72.4
378	77	78.6
379	72	73.5
380	74	75.5
381	72	73.5
382	79	80.6
383	75	76.5
Average	72.5 ± 4.4	74.0 ± 4.7

All implanted rats recovered well from surgery, and no clinical observational endpoints were reached, allowing all 10 animals to continue until the end of the study.

**Figure 2:**
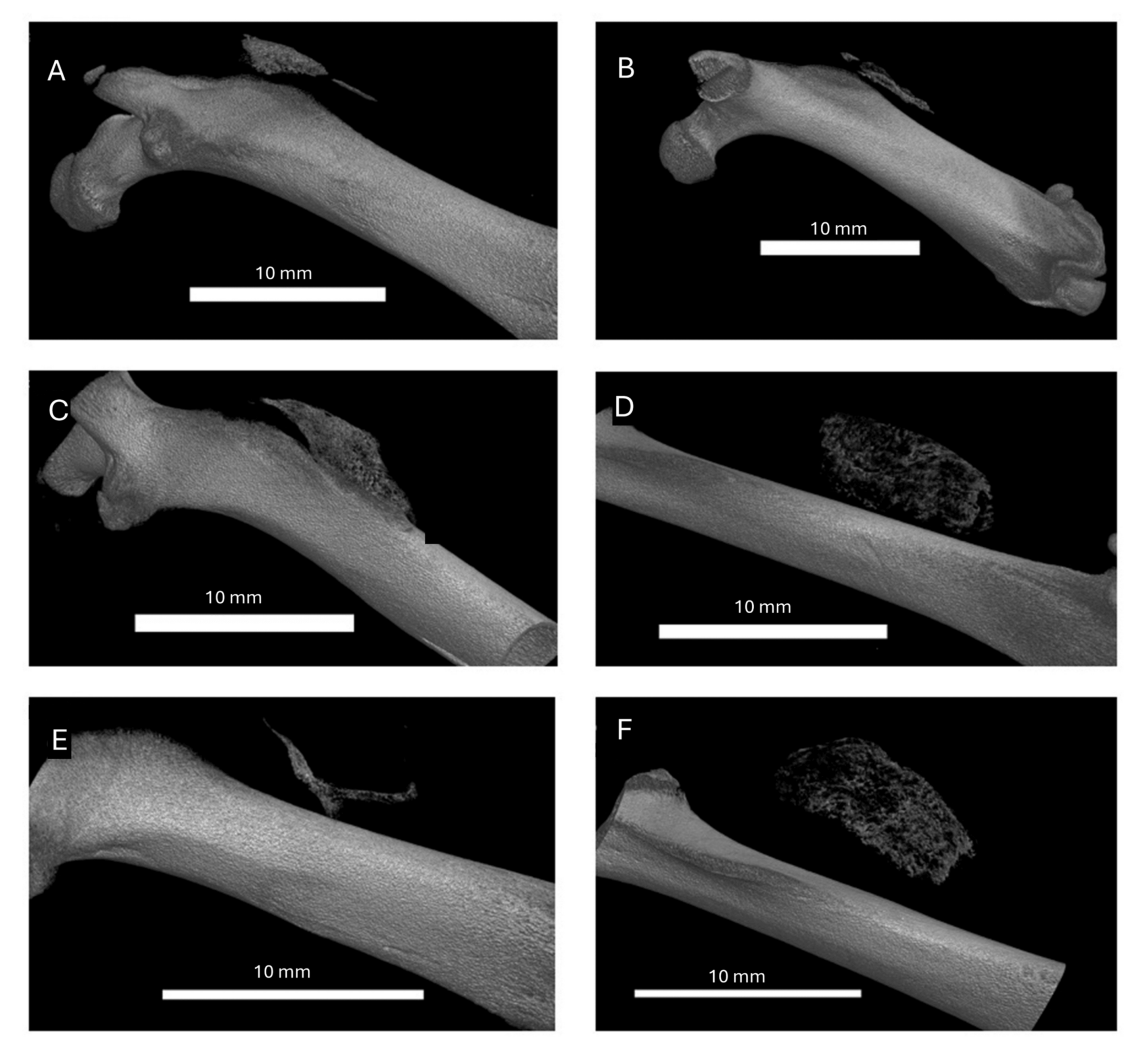
Representative three-dimensional micro-CT reconstructions of the left and right legs for each animal (A) Animal 375, SRO. (B) Animal 375, ACS. (C) Animal 379, SRO. (D) Animal 379, ACS. (E) Animal 382, SRO. (F) Animal 382, ACS. All scale bars = 10 mm. SRO: supernatant run-off.

**Figure 3:**
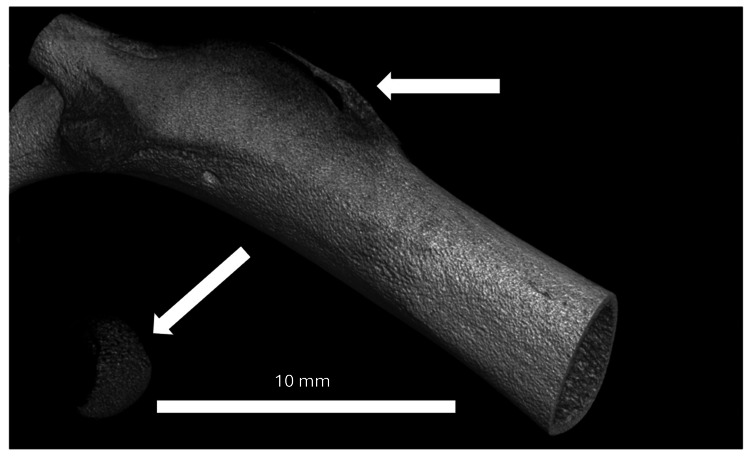
Three-dimensional micro-CT reconstruction of the left leg of animal 377, showing that the SRO resulted in the formation of mineralised tissue in two distinct regions White arrows indicate the mineralised structures. Scale bar = 10 mm. SRO: supernatant run-off.

In both legs, implanted with the collagen sponge or the run-off liquid, micro-CT identified radiopaque mineralised foci, as depicted in Figure 2; these were quantified by threshold-based segmentation and subsequently phenotyped histologically to distinguish bone from other mineralised tissues. For one rat (377), depicted in Figure 3, two distinct areas of mineralised tissue structures in two different planes were observed in the left leg (leg with SRO). Beyond the two discrete foci noted in animal 377, no animals showed evidence of mineralisation at sites distant from the injection/implantation site. Total mineralised volume per animal was calculated by summing the volumes of all discrete mineralised foci within the injection site. Representative images in Figure 2 show the three-dimensional micro-CT image reconstructions of three animals, displaying a larger tissue volume for the ACS implants than for the SRO implants. 

Micro-CT analysis

**Figure 4:**
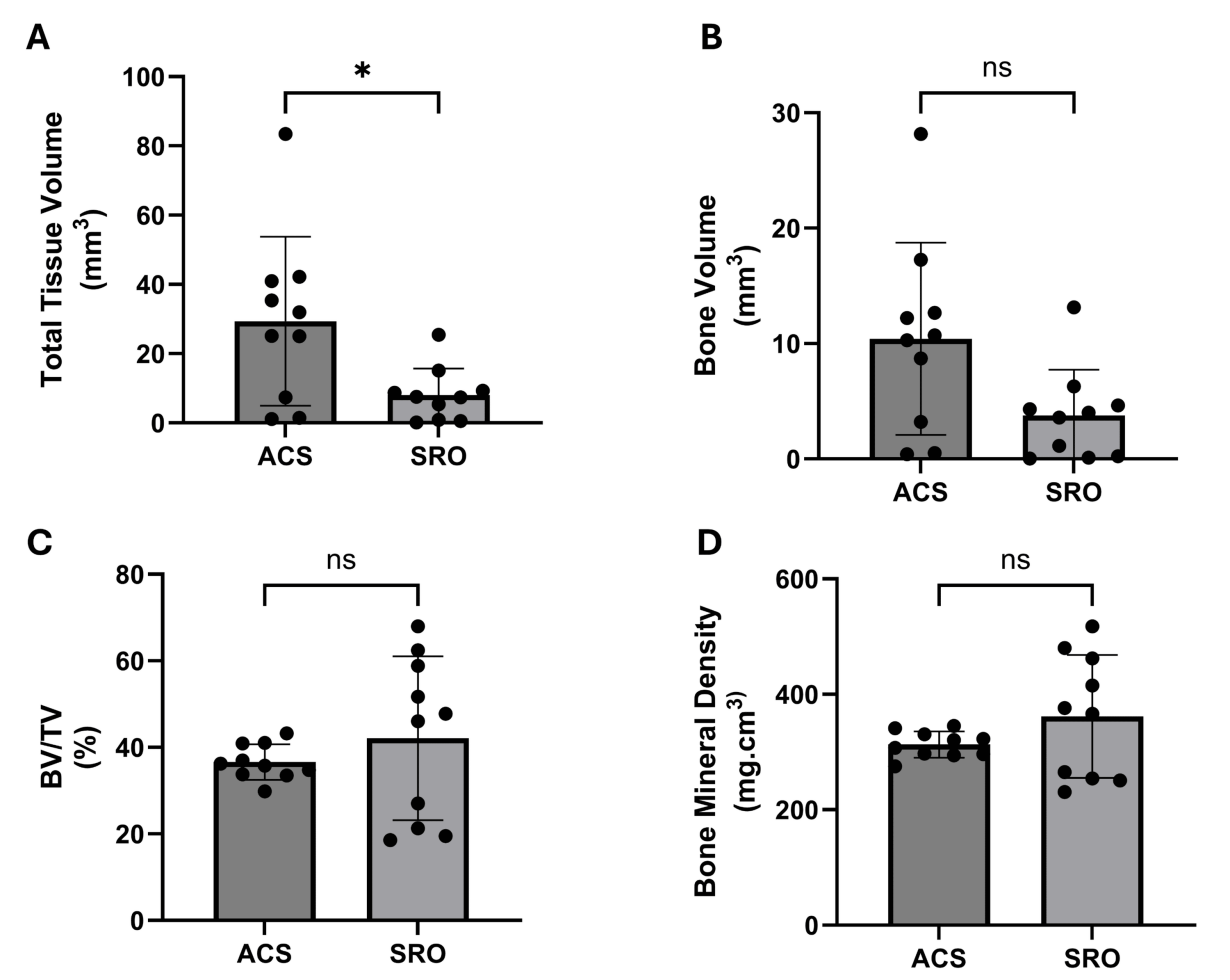
Quantitative micro-CT analysis of mineralised tissue formation in ACS and SRO groups at 28 days Each animal received an absorbable collagen sponge (ACS) soaked with rhBMP-2 solution in the right hindlimb and the corresponding supernatant run-off (SRO) collected after centrifugation in the left hindlimb. (A) Total tissue volume (TV). (B) Bone volume (BV). (C) Bone volume fraction (BV/TV). (D) Bone mineral density (BMD). Data are presented as mean ± SD (n = 10 per group). Statistical comparisons were performed using the Mann-Whitney Test. *p < 0.05; (A) p = 0.0288, Mann-Whitney U = 21, (B) p = 0.0630, Mann-Whitney U = 25, (C) p = 0.4813, Mann-Whitney U = 40, (D) p = 0.4813, Mann-Whitney U = 40.

Total tissue volume was significantly greater in the ACS group compared with the SRO group (29.4 ± 24.4 mm³ vs 8.05 ± 7.69 mm³; BV, p = 0.0288, Mann-Whitney U = 21; Figure 4a), with a large effect size (r = 0.70). Absolute bone volume showed a similar trend favouring the ACS group (10.4 ± 8.3 mm³ vs 4.2 ± 4.3 mm³), though this difference did not reach statistical significance (p = 0.0630, Mann-Whitney U = 25; Figure 4b).

Bone volume fraction (BV/TV) (ACS: 36.6 ± 4.1% vs SRO: 42.0 ± 18.8%; p = 0.4813, Mann-Whitney U = 40; Figure 4c) and bone mineral density (ACS: 313,111 ± 22,733 mg/cm³ vs SRO: 361,874 ± 106,448 mg/cm³; p = 0.4813, Mann-Whitney U = 40; Figure 4d) did not differ significantly between groups. There was no correlation between SRO volume administered (Table 1) and subsequent TV, BV, or BV/TV measurements. 

Histology

Histological evaluation revealed that mineralised tissue formation occurred in both groups, with heterogeneity in tissue identity and in the stages of architectural maturation between samples. Not all mineralised foci identified by micro-CT corresponded to histologically confirmed bone.

**Figure 5:**
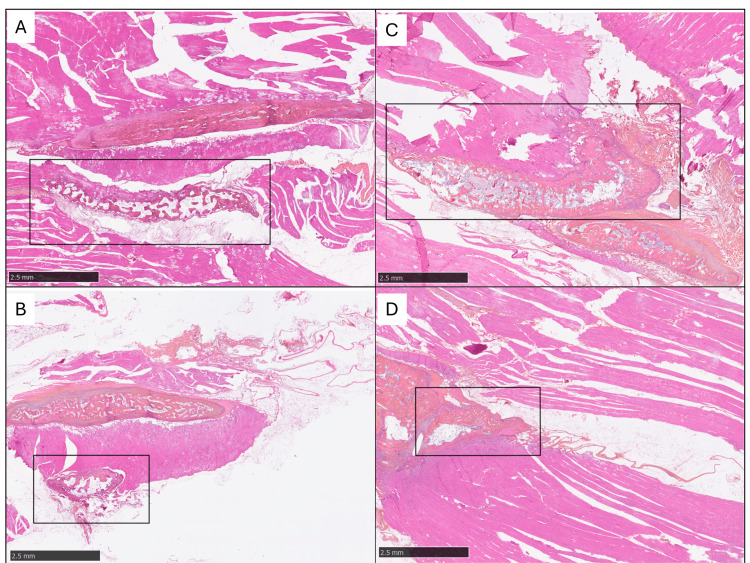
Images of sections from SRO implant sites stained with haematoxylin and eosin Implantation sites highlighted by black rectangles. Scale bar 2.5 mm. A = 374L, B = 375L, C = 379L, and D = 380L. SRO: supernatant run-off.

Histological assessment of the SRO group revealed variable tissue phenotypes underlying the micro-CT mineralisation (Figure 5). The degree of structural maturation at Day 28 varied considerably across the cohort, with mineralised foci ranging from predominantly mineralised cartilage to well-circumscribed ectopic ossicles with bone formation, illustrating the sequential stages of BMP-2-induced endochondral ossification (Figure 5). 

**Figure 6:**
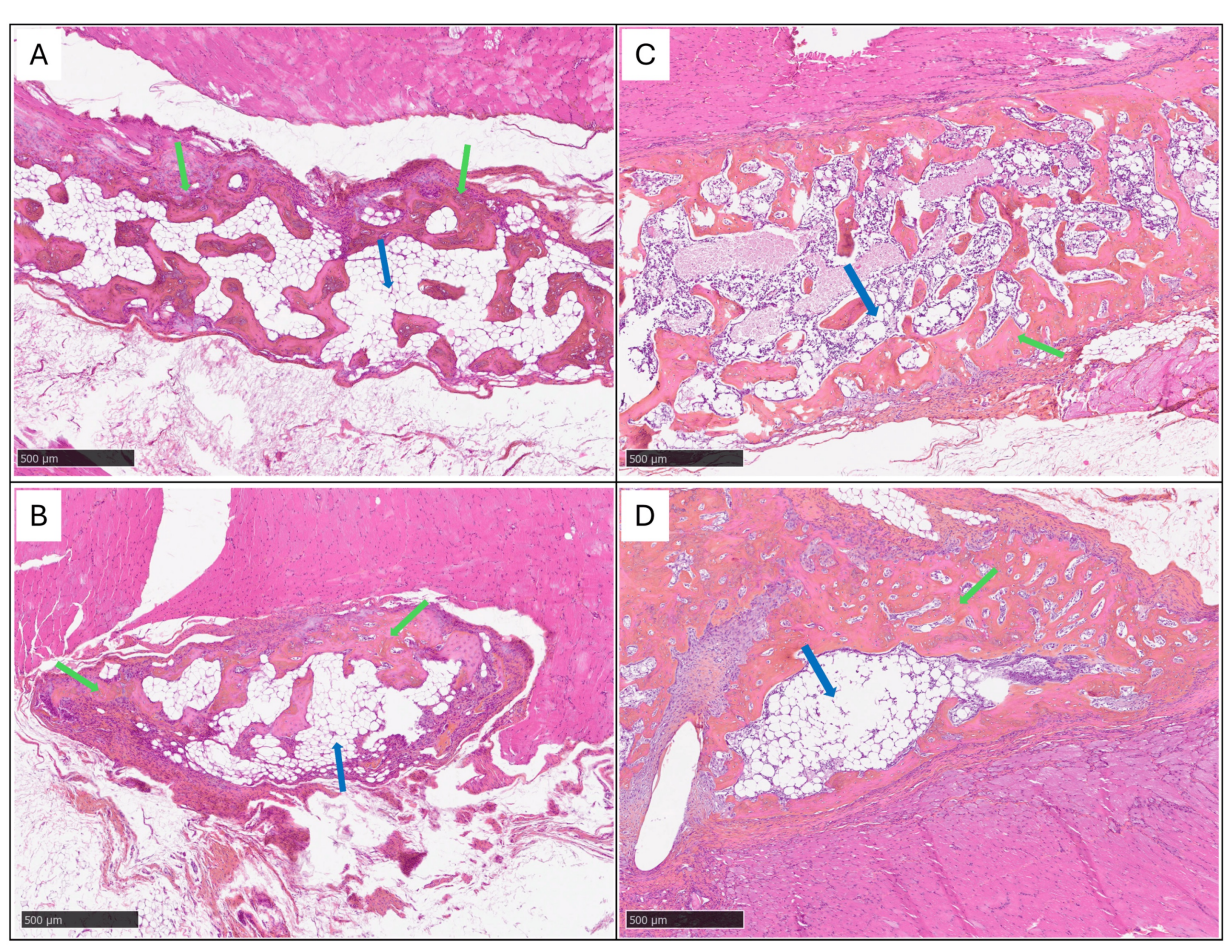
Histological sections of SRO implant sites stained with haematoxylin and eosin Scale bar = 0.25 mm. (A) 374L, (B) 375L, (C) 379L, and (D) 380L. Green arrows indicate examples of woven bone trabeculae. Blue arrows indicate examples of marrow spaces. SRO: supernatant run-off.

Some SRO animals presented with a predominantly chondrogenic phenotype, characterised by a solid core of hypertrophic cartilage surrounded by a mineralising cortical rim (e.g. 375L, 380L). In these specimens, the micro-CT mineralisation corresponded primarily to mineralised cartilage rather than bone. These samples provided histological evidence that the soluble SRO factor can initiate the endochondral pathway, though progression to mature bone was incomplete at this time point (Figure 6B and D).

In contrast, other animals in the SRO group exhibited more advanced remodelling, demonstrating a corticocancellous-like architecture (e.g. 374L, 379L). In these specimens, the cartilaginous template had been substantially replaced by trabecular bone with osteocyte-containing lacunae and osteoblast lining, and a central medullary cavity was established. These cavities contained marrow-like spaces with adipocyte-rich stromal tissue and occasional cellular aggregates consistent with early marrow development (Figure 6A and C). These findings indicate that dissociated rhBMP-2 can induce bone formation in a subset of animals, though the proportion of SRO sites showing histologically confirmed bone versus mineralised non-osseous tissue should be noted. 

**Figure 7:**
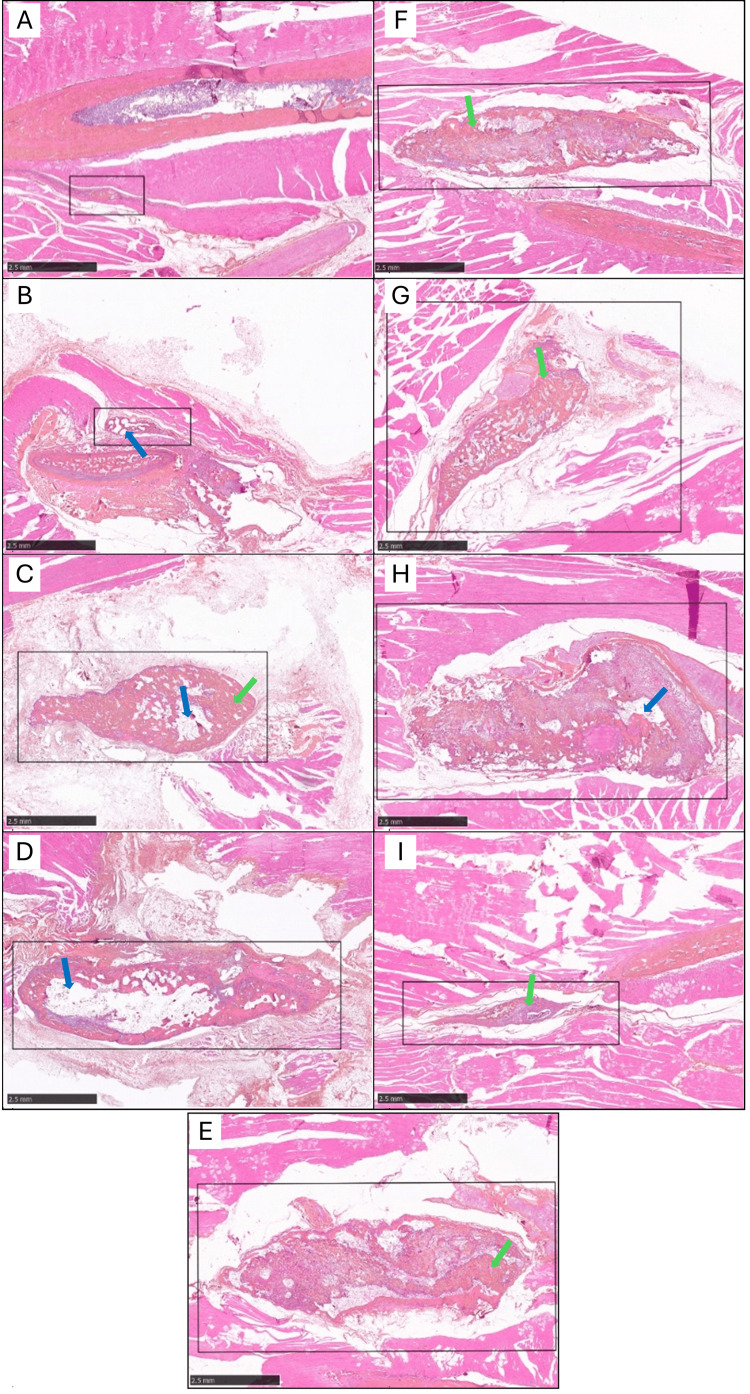
Histological sections of ACS implant sites stained with haematoxylin and eosin Scale bar = 2.5 mm. (A) 374R, (B) 375R, (C) 376R, (D) 377R, (E) 379R, (F) 380R, (G) 381R, (H) 382R, and (I) 383R. Green arrows indicate examples of dense networks of trabeculae. Blue arrows indicate examples of early marrow spaces. ACS: absorbable collagen sponge.

**Figure 8:**
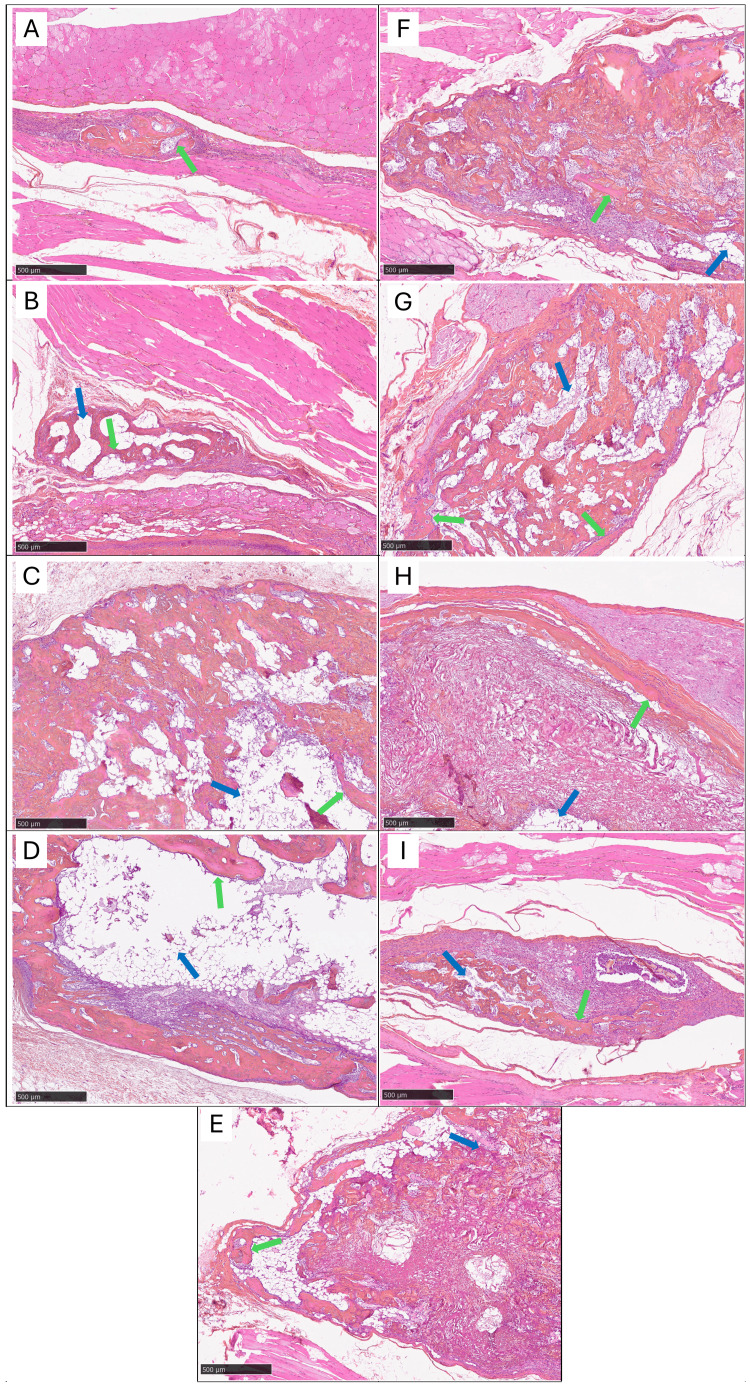
Histological sections of ACS implant sites stained with haematoxylin and eosin Scale bar = 0.5 mm. (A) 374R, (B) 375R, (C) 376R, (D) 377R, (E) 379R, (F) 380R, (G) 381R, (H) 382R, and (I) 383R. Green arrows indicate examples of osteoblasts lining woven bone trabeculae containing osteocytes within lacunae. Blue arrows indicate examples of marrow spaces with vascular invasion. ACS: absorbable collagen sponge.

In the ACS samples (Figures 7, 8), all rhBMP-2-treated intramuscular sites, except 378R, demonstrated well-formed ectopic ossicles composed of maturing woven bone that could be identified by histological analysis. Across the specimens, the ossicles were sharply demarcated from the surrounding skeletal muscle by a thin, collagen-rich fibrous capsule, with only minimal chronic inflammatory infiltrates and no detectable residual implant material. Most animals exhibited residual cartilage cores or chondrocyte-containing transition zones indicative of ongoing cartilage-to-bone replacement. Despite this shared overall architecture, the ossicles displayed meaningful inter-animal variation in internal organisation, particularly in the balance between bone density and marrow development. 

ACS animals 376R (Figure 7C) and 377R (Figure 7D) exhibited a marrow-dominant phenotype, characterised by large adipocyte-rich cavities and expanded intertrabecular spaces. These specimens contained extensive early marrow fat and, in some regions, cyst-like voids formed by confluent adipocytes and stromal tissue. In contrast, most animals in this group demonstrated bone-dominant phenotypes, with denser trabecular networks, smaller stromal compartments, and more prominent residual cartilage undergoing endochondral ossification. Marrow development in these animals was primarily fibrovascular, with only scattered adipocytes.

Across all animals, woven bone trabeculae contained numerous osteocytes and were frequently lined by osteoblasts depositing osteoid, reflecting ongoing osteogenesis. Osteoclasts were rare, consistent with limited remodelling at this early stage. Vascular invasion was evident throughout the marrow spaces, supporting active ossification and early marrow development. Together, these findings confirm the robust osteoinductive activity of rhBMP-2 and reveal a biologically plausible spectrum of outcomes ranging from highly adipose, marrow-filled ossicles to more compact, bone-dense structures with persistent cartilage templates. 

## Discussion

This study demonstrates that rhBMP-2, once detached from its carrier matrix, retains the capacity to induce ectopic mineralisation with variable histological phenotypes. We showed that centrifuged supernatant liquid (SRO) induced mineralised tissue formation in all animals, though the histological identity ranged from mineralised cartilage to marrow-containing ossicles, with a subset of animals exhibiting corticocancellous-like architecture with bone formation at 28 days.

In previous studies, Hsu et al. [9] reported no bone formation when applying the supernatant of mildly compressed ACS in vivo. However, we showed that centrifuged supernatant liquid (SRO) containing dissociated BMP-2 progresses towards bone formation in a manner similar to BMP-2 + ACS at intramuscular ectopic sites. In Hsu et al., 0.2 mL of 0.43 mg/mL was injected without ACS, providing a total quantity of rhBMP-2 per site of 86 µg. This significantly exceeds the dose within the SRO implanted in this study, which was estimated, based on in vitro data, to have a mean of 16.4 µg. One possible explanation is the location of the implant. Studies have shown that both intramuscular and intermuscular delivery of rhBMP-2 consistently produce more robust and earlier ectopic bone formation than subcutaneous delivery [27,28]. The richer vascular supply of intramuscular and intermuscular sites provides greater oxygen tension, nutrient delivery, and recruitment of circulating osteoprogenitor cells, all of which enhance rhBMP-2-induced osteogenesis [27,28]. Furthermore, skeletal muscle harbours abundant mesenchymal stem/progenitor cells (e.g. muscle satellite cells or fibro-adipogenic progenitors) that readily differentiate into osteoblasts under BMP-2 stimulation [27,29]. By contrast, subcutaneous tissue (rich in adipose tissue) is less vascularised and contains fewer inducible osteogenic precursors, which slows and diminishes bone formation despite BMP-2 exposure [27].

The ACS group exhibited greater total tissue volume; this likely reflects the presence of the scaffold itself and the significantly higher proportion (89% ± 6%) of the rhBMP-2 content following centrifugation. BV/TV and BMD did not differ significantly between groups, but these parameters reflect mineralisation density and fraction, not histological bone quality. Histology demonstrated variable tissue identity underlying the radiopacity, particularly in the SRO group, where mineralised foci ranged from mineralised cartilage to mature bone. Nevertheless, the presence of mineralised tissue in all SRO animals indicates that rhBMP-2 alone, once detached from the scaffold, retains the capacity to induce ectopic mineralisation with osteogenic potential.

The SRO and ACS group intramuscular implants exhibited broadly similar overall patterns of BMP-2-induced heterotopic ossification at four weeks, including woven bone, active osteogenesis, thin fibrous encapsulation, and minimal chronic inflammation. However, some systematic differences emerged between series. SRO-series ossicles tended to develop more uniformly mineralised trabeculae with occasional early lamellar organisation and predominantly fibrovascular or mixed haematopoietic marrow. In contrast, several ACS-series ossicles, particularly 376R and 377R, demonstrated markedly expanded adipocyte-rich marrow cavities, producing large stromal voids and a more cystic internal architecture. Endochondral remnants were frequent in both series, though more consistently preserved in SRO-series samples, whereas some ACS-series ossicles lacked identifiable cartilage, likely reflecting accelerated tissue turnover or replacement by adipose marrow. Overall, the SRO series displayed a pattern of structural consolidation with moderate marrow maturation, whereas the ACS series showed greater heterogeneity, with several animals exhibiting advanced marrow adipogenesis despite less mature bone architecture.

These contrasting trajectories may well be driven by the substantial difference in localised rhBMP-2 dose (approximately 11% in SRO vs 89% in ACS). The ACS group, which retained the majority of the osteoinductive load, exhibited a predominantly "hyper-productive" osteogenesis. For example, implant 380R presented as dense, sclerotic nodules. In this sample, the deposition of woven bone and persistent hypertrophic cartilage occupied the entire implant volume, effectively excluding the formation of a marrow cavity. This "hard callus" phenotype aligns with findings by Zara et al. [30], who reported that supraphysiological concentrations of rhBMP-2 (150-600 μg/mL), well below the doses in this study (1.5 mg/mL pre-centrifugation), can paradoxically delay structural maturation while promoting a prolonged, disorganised rapid-growth phase.

Furthermore, the variability within the ACS group included two distinct pathological features absent in the SRO group, specifically cyst formation and adipogenic dominance. For example, animal 377R presented with a large, hollow cystic structure bounded by a cortical shell, while animal 376R consisted almost entirely of adipose tissue with minimal trabecular bone. These findings are consistent with malformations reported in other preclinical models using high-dose rhBMP-2. Pan et al. [22] observed the formation of cyst-like osteolytic defects and adipose-filled voids in sheep treated with rhBMP-2 at 1.5 mg/cc. Additionally, the adipose-dominant phenotype observed in animal 376R supports the known mechanism of BMP-2 signalling overlap with the adipogenic pathway. High doses of BMP-2 have been shown to upregulate peroxisome proliferator-activated receptor-gamma [7] (PPARγ), a master regulator of adipogenesis, shifting mesenchymal stem cell differentiation away from osteoblasts and towards adipocytes. The presence of these cystic and adipogenic phenotypes exclusively in the ACS group suggests a dose relationship, consistent with other studies [6,22]. Higher doses, while efficient for total bone volume, create a supraphysiological microenvironment that increases the risk of structural abnormalities.

Previous studies have established that rhBMP-2 interacts avidly with extracellular matrix (ECM) components, specifically heparan sulphate and fibronectin, through its N-terminal heparin-binding domain [31-34]. In the absence of an exogenous scaffold (ACS), we hypothesise that the host muscle fascia acts as a substitute biological scaffold, sequestering the soluble rhBMP-2. However, the density of these binding sites within the host tissue is finite. In the SRO group, the lack of a scaffold meant that osteoinduction was reliant on the containment of the fluid by the host fascia and, when retained, physiological bone formed. However, we speculate that once the local ECM binding capacity is saturated, any excess soluble rhBMP-2 is likely subject to rapid clearance via interstitial bulk flow and diffusion, as described by Doan et al. [20] and Cowin [21]. We hypothesise that this saturation effect may also explain why larger liquid volumes of SRO (Table 1) did not result in proportionally larger ossicles, as the "effective" osteoinductive dose was constrained by the availability of local retention sites rather than the total injected volume.

Conversely, the ACS group demonstrates that while high-dose BMP-2 implanted on ACS guarantees bone formation, it may "lock" the tissue in an immature or pathological state because of sustained osteogenic and adipogenic overstimulation. This validates the clinical relevance of the "burst release" model: even without a scaffold, leaked rhBMP-2 is not inert; it is capable of forming marrow-containing bone, potentially contributing to the ectopic ossification sequelae observed in spinal fusion complications.

These findings have potentially significant translational implications. In clinical settings, BMP-2 applied on ACS is known to dissociate rapidly, creating a risk for off-target migration. Our data provide direct experimental evidence that even small amounts of liberated BMP-2 can induce ectopic mineralised tissue at sites away from the carrier. This supports the concerns raised by clinical and preclinical reports of ectopic ossification and neural impingement following high-dose BMP-2 administration. Based on non-human primate BMP-2 retention data, our findings could represent a conservative estimate of the amount of BMP-2 initially released. The non-human primate data report approximately five times higher rhBMP-2 release (53% of the implanted dose) in the first day [4] than was tested here (approximately 11%; Figure 1); thus, the risk of ectopic mineralisation in a human clinical scenario may be further increased. Importantly, the clinical relevance of ectopic mineralisation does not depend solely on whether histology confirms mature bone. Mineralised tissue of any composition, whether bone, mineralised cartilage, or other mineralised matrix, formed in proximity to neural elements could contribute to symptoms via mass effect, mechanical tethering, reduced tissue compliance, or associated local inflammatory and fibrotic responses. Therefore, risk assessment for "ectopic mineralisation" following rhBMP-2 administration should not rely solely on whether the mineralised tissue meets histological criteria for bone.

Study limitations

As noted above, in the in vitro part of this study, there was a moderate but non-significant Pearson correlation (0.47) between the volume of liquid removed (average 64.6 µL) in the centrifugation step and the amount of rhBMP-2 in the SRO. The amount of liquid removed in the in vivo phase was higher (72.5 µL), and therefore the amount of rhBMP-2 in the SRO group may have been correspondingly higher, although no correlation between SRO volume and bone mineral measurements was found.

A further limitation is that rhBMP-2 concentration was not directly quantified in the SRO samples used for the in vivo phase. The 11.3% ± 6% release estimate was derived from the parallel in vitro experiment and applied to the in vivo samples based on the assumption of methodological equivalence. Individual variability in protein binding and release kinetics means that the actual rhBMP-2 dose delivered to each animal's SRO site remains an estimate rather than a direct measurement. This may partly explain the lack of correlation between SRO volume and the resulting bone formation observed in vivo.

This study was performed in rats, which have a faster bone repair rate than humans [35], and thus caution should be exercised when translating the bone formation, the required total dose, and the dose concentrations (mg/cc) between species.

Additionally, the 28-day endpoint, while sufficient to demonstrate osteoinductive potential, precedes complete bone maturation. Thus, the persistent immature chondroid-to-osteoid histology observed in some samples may progress to complete trabecular organisation at later time points. The clinical concentration of rhBMP-2 (1.5 mg/mL) used in this study, while reflecting real-world surgical practice, represents supraphysiological dosing that has been associated with delayed bone maturation and structural abnormalities in other models [30]. The reviewer of the micro-CT images was not blinded to the groups.

## Conclusions

Surface-attached rhBMP-2 leads to a rapid release of rhBMP-2 in vivo, which is then vulnerable to migration caused by interstitial flow. Liberated rhBMP-2 may be carried to proximal sites. In this study, we demonstrate that even dissociation of approximately 11% of rhBMP-2 can induce ectopic mineralised tissue, which on histology ranges from mineralised cartilage to mature bone.

In anatomically constrained clinical settings, even mineralised tissue that does not meet histological criteria for bone may be clinically relevant if formed adjacent to neural elements. These findings offer new insight into the progression of ectopic mineralisation using surface-attached rhBMP-2 delivery vehicles and underscore the need for improved controlled-release strategies.
